# *Artemisia absinthium* L. Extract Targeting the JAK2/STAT3 Pathway to Ameliorate Atherosclerosis

**DOI:** 10.3390/foods14132381

**Published:** 2025-07-05

**Authors:** Jiayi Yang, Tian Huang, Lijie Xia, Jinyao Li

**Affiliations:** Xinjiang Key Laboratory of Biological Resources and Genetic Engineering, College of Life Science and Technology, Xinjiang University, Urumqi 830017, China; 107552301107@stu.xju.edu.cn (J.Y.); 107552103422@stu.xju.edu.cn (T.H.)

**Keywords:** terpenoids, *Artemisia absinthium* L., extraction and purification, atherosclerosis

## Abstract

*Artemisia absinthium* L. contributes to ecological stabilization in arid regions through its deep root system for sand fixation and soil microenvironment modulation, thereby effectively mitigating desertification. Total terpenoids have been extracted from *A. absinthium* (AATP) and found to have antioxidant and anti-inflammatory activities. Terpenoids are a class of natural products derived from methyl hydroxypropanoic acid, for which their structural units consist of multiple isoprene (C5) units. They are one of the largest and most structurally diverse classes of natural compounds. However, there are still large gaps in knowledge regarding their exact biological activities and effects. Atherosclerosis (AS) is a prevalent cardiovascular disease marked by the chronic inflammation of the vascular system, and lipid metabolism plays a key role in its pathogenesis. This study determined the extraction and purification processes of AATP through single-factor experiments and response surface optimization methods. The purity of AATP was increased from 20.85% ± 0.94 before purification to 52.21% ± 0.75, which is 2.5 times higher than before purification. Studies have shown that the total terpenoids of *A. absinthium* significantly reduced four indices of serum lipids in atherosclerosis (AS) rats, thereby promoting lipid metabolism, inhibiting inflammatory processes, and hindering aortic wall thickening and hepatic fat accumulation. It is known from network pharmacology studies that AATP regulates the Janus kinase/signal transducer (JAK/STAT) signaling axis. Molecular docking studies have indicated that the active component of AATP effectively binds to Janus kinase (JAK2) and signal transducer (STAT3) target proteins. The results indicate that AATP can inhibit the release of pro-inflammatory mediators (such as reactive oxygen species (ROS)) in LPS-induced RAW264.7 macrophages. It also inhibits the M1 polarization of RAW264.7 macrophages. Protein immunoblotting analysis revealed that it significantly reduces the phosphorylation levels of Janus kinase (JAK2) and the signal transducer and activator of transcription 3 (STAT3). Research indicates that the active components in *A. absinthium* may exert anti-atherosclerotic effects by regulating lipid metabolism and inhibiting inflammatory responses. It holds potential value for development as a functional food or drug for the prevention and treatment of atherosclerosis.

## 1. Introduction

Cardiovascular disease is one of the leading causes of mortality worldwide [1]. Atherosclerosis (AS), a prevalent cardiovascular disorder, develops through chronic lipid-driven vascular inflammation [2]. It serves as the primary pathological basis for multiple cardiovascular conditions, including myocardial infarction and acute coronary syndromes [3,4]. AS was recognized as a widespread and life-threatening disease that could lead to severe clinical outcomes and pose substantial public health risks [5]. Therefore, the prevention and treatment of atherosclerosis are considered crucial medical priorities. The mechanisms underlying the initiation and progression of atherosclerosis were complex and involved multiple pathological processes, including inflammatory responses, dysregulated lipid metabolism, oxidative stress, endothelial dysfunction, and neointimal hyperplasia [6,7]. The pathological process is initiated with vascular endothelial injury. Under various pathogenic stimuli, multiple inflammatory mediators activate endothelial cells, triggering the secretion of monocyte chemoattractant proteins that subsequently induce monocyte recruitment. These cells differentiate into macrophages, which phagocytose modified LDL particles, accumulate lipids, and ultimately transform into cholesterol-laden foam cells. Cumulatively, it causes vascular injury and accelerates atherogenesis [8,9,10,11]. From the initial lesion to plaque formation, the inflammatory response is always involved and drives the entire pathologic process of atherosclerosis. The pathological progression of atherosclerosis is characterized by the aberrant activation of inflammatory cascades and the massive infiltration of lipid-laden macrophages, which constitute the hallmark pathological features. Lipid and cholesterol deposition in the vascular system, coupled with elevated circulating inflammatory factors, trigger localized inflammatory responses [12]. However, existing therapeutic strategies for atherosclerosis exhibit significant limitations, and treatment options with minimal adverse effects remain inadequate.

*A. absinthium* is extremely drought-, heat-, and cold-tolerant and is widely distributed in wild wasteland and grassland habitats [13]. *A. absinthium* is described in the pharmacopeias of many countries under different names, which vary depending on the country in question. It was a commonly used medicinal plant in various regions, including Europe and Central Asia, for the treatment of hepatitis, dyspepsia, stomach pain, anemia, and anorexia [14]. *A. absinthium* is the principal component of absinthe, a beverage consumed in Europe for approximately 300 years. It was thought to have properties that stimulated the appetite, promoted digestion, and nourished the body [15,16]. *A. absinthium* is rich in bioactive components, including total terpenoids, flavonoids, and polyphenols. These ingredients have cardiovascular, anti-inflammatory, and antioxidant properties. The chemical composition and bioactivity of ethanol extracts obtained primarily from *A. absinthium* leaves and stems were investigated to enhance the understanding of the phytochemistry and bioactivity of this ancient medicinal plant with significant medicinal potential. To this end, the ethanol extracts of the leaves and stems were analyzed using liquid chromatography–mass spectrometry (LC-MS), thermal analysis (TG-DSC), and Fourier transform infrared spectroscopy (FT-IR). The anti-inflammatory effects of the extracts were tested in a mouse ear edema model [17,18,19]. But which group of substances in absinthe exerts this anti-inflammatory effect, through what pathways, and whether they inhibit the progression of atherosclerosis remain unknown. Finally, how these substances are extracted is also unclear. These questions are worth investigating by the authors.

Terpenoids are among the largest and most structurally diverse compounds in nature. Terpenoids are relatively common compounds found in natural products. They are critical to the physiological processes, growth, and developmental capacity of plants [20]. Recent research data reveal that the pharmacological value of medicinal plant-derived terpenoids is becoming increasingly prominent, and their potential for clinical applications is being continuously explored. They prevent and treat cardiovascular disease, and they are anti-inflammatory and hypoglycemic [21,22,23]. Terpenoids are widely distributed in various natural herbs and have the potential to become natural medicinal drugs [24]. Studies have shown that terpenoids, particularly terpenoids, can treat cardiovascular diseases by reducing oxidative stress and activating the Nrf2/HO-1 pathway [24]. Apigenin, 2-methyl-5-(1-methylethyl)phenol, is the main component of essential oils from the Labiatae family [25]. Before apigenin was studied, it was generally considered a safe additive and flavoring agent. Subsequent studies have demonstrated that apiole exhibits broad protective properties against disease states, including inflammation, oxidative stress, tumor growth, and microbial infections [26]. Vascular smooth muscle cell (VSMC) migration and proliferation are key processes in neointimal formation during atherosclerosis [27]. Studies have shown that apigenin inhibits PDGF-BB-induced RASMC (vascular smooth muscle cell) migration and proliferation, suppresses PDGF-BB-induced MAPK phosphorylation, and also inhibits the increased sprouting growth of aortic strips [28].

Experimental design enhancement has significantly utilized the response surface methodology (RSM) [29]. This optimization method defines the functional relationship between several parameters and their related response values using numerous quadratic regression equations. By examining these regression equations, the optimal process parameters were determined and predicted using Ordel [30]. This method offered several advantages, such as short experimental duration, ease of use, and high accuracy and predictive power. At that time, it was used in the process optimization of chemical and food products [31,32].

Cyberpharmacology systematically predicts complex interactions between drugs and multiple targets by constructing networks of biomolecular interactions [33] and elucidating the latent complexity relationships between diseases, compounds, and protein targets for drug discovery and development efforts [34]. Currently, network pharmacology analysis mainly uses the chemical constituents of Chinese medicines in databases for target prediction and functional analysis. The chemical composition data obtained through measurement will be more realistic and reliable once network pharmacology analysis is conducted [35]. Network pharmacology methods provide an effective way to analyze the effects of complex herbal medicines [36]. The drug design technique of molecular docking involves connecting a receptor macromolecule to the three-dimensional structure of a ligand compound [37]. To further understand the molecular relationships between TCM active ingredients and illness targets, the small-molecule components of TCM will be molecularly docked with target proteins [38]. The mutual corroboration of computational predictions and experimental results greatly enhances the credibility of the conclusions on the drug’s mechanism of action [39,40]. The present study rationally combines predictive techniques and a rational experimental design to explore the possible pathways of drug action.

Compared with the traditional one-factor method, the one-factor experiment combined with the BBD experimental design was optimized for the extraction process of AATP, and a more ideal enrichment effect was obtained with the graded purification of macroporous resin. Current treatments mainly focus on insufficient intervention in the early stages of advanced atherosclerosis. Obesity exacerbates some risk factors for atherosclerosis [2]. Even though there are now many treatments available for atherosclerosis, their high costs prevent many people from choosing the best treatment [6]. The disease has a high recurrence rate, and progress in later-stage treatment is slow, urgently requiring the development of highly effective drugs with preventive properties. This paper innovatively combines pharmacological predictions from a combined network with experimental pharmacology to investigate the therapeutic effects of AATP against anti-atherogenicity. This investigation provides a new theoretical strategy for analyzing the pathological mechanisms of atherosclerosis and developing therapeutic strategies with fewer side effects.

## 2. Materials and Methods

### 2.1. Materials and Reagents

*A. absinthium* powder was provided by Xinjiang Urumqi Tianyifeng Biological Technology Co., Ltd., Urumqi, Xinjiang, China, courtesy of Xinjiang Baokang Pharmaceutical Co. OX-LDL was provided by Medical Source Biotechnology (Guangzhou, China). LPS (L8880) was purchased from Beijing Solarbio Science & Technology Co. Simvastatin was purchased from Shanghai Yuanye Biotechnology Co. Total cholesterol (TC) (A111-1-1), triglycerides (TGs) (A110-1-1), low-density-lipoprotein cholesterol (LDL-C) (A112-1-1), and high-density-lipoprotein cholesterol (HDL-C) (A113-1-1) assay kits were purchased from Nanjing Jianche Bioengineering Institute (Nanjing, China), in addition to rabbit anti-mouse JAK2, STAT3, p-JAK2, and p-STAT3.

### 2.2. Extraction Process of AATP

The total terpenoids of *A. absinthium* were extracted by adding *A. absinthium* powder to distilled water or different concentrations of ethanol, depending on the extraction temperature and number of extractions. The filtrate is then swirled at 8000× *g* for 10 min. The liquid was reacted with a rotary evaporator at 60 °C under vacuum.

#### 2.2.1. A One-Way Experiment to Extract AATP

For this, the total terpenoids are used as an indicator. A univariate experimental design was applied to examine the influence of water and ethanol concentrations (45, 65, 75, 80, and 95%), solid–liquid ratios (1:10, 1:15, 1:20, 1:25, and 1:30 g/mL), extraction times (20, 30, 40, 50, 60, 60, and 70 min), extraction temperatures (40, 50, 60, 70, 80, and 90 °C), and number of extractions (1, 2, 3, and 4) on the total terpenoids. The best conditions for each factor were found. In a single-factor experiment, a single factor is altered in each instance, while the remaining factors remain constant. The optimal extraction combination is ultimately determined by selecting optimal conditions at three levels of each factor for response surface design.

#### 2.2.2. Response Surface Experimental Design for the Extraction of AATP

The material–liquid ratio (A), extraction temperature (B), and extraction duration (C)—the three independent variables—were selected as critical parameters for optimizing absinthe terpenoid yields based on single-factor experimental results. The data analysis of the response surface optimization was conducted using the Design Expert 13 software, resulting in the generation of a three-factor, three-level model comprising 17 experimental groups.

### 2.3. Purification Using Macroporous Adsorption Resin

#### 2.3.1. Static Sorption and Desorption Experiments for Macroporous Resin-Type Screening

After soaking in 95% ethanol for 24 h, macroporous resins D101, AB-8, NKA-9, DM130, and X-5 were repeatedly rinsed with distilled water until they were clean of any ethanol smell. After soaking in a 5% HCl solution for five hours, pure water was used to rinse the resin, which was then inserted in a 5% NaOH solution for five hours and then rinsed again with distilled water until the effluent’s pH was neutral. For subsequent usage, it was allowed to dry at ambient temperature [41].

The five macroporous resins were weighed at 2.0 g and combined with 20 mL of AATP in 100 mL Conical flasks. Filtration resin and AATP content in the filtrate were determined. Using the same oscillation parameters and adsorption conditions, the saturated resin was desorbed with 40 mL of aqueous 95% ethanol solution, the resin was filtered, and the AATP content of the filtrate was determined. The adsorptive and adsorbent property rates for each packing material were calculated using the following equations:

$$\mathrm{sorption}\;\mathrm{capability}\;(\mathrm{mg}/g)\;Q_{e}=\frac{(C_{0}-C_{e})V_{1}}{m}$$

$$\mathrm{sorption}\;\mathrm{ratio}\;(\%)\;A=\frac{\left(C_{0}-C_{e}\right)}{C_{0}}\times 100\%$$

$$\mathrm{desorption}\;\mathrm{capability}\;(\mathrm{mg}/g)\;Q_{d}=\frac{C_{2}V_{2}}{m}$$

$$\mathrm{desorption}\;\mathrm{ratio}\;(\%)\;D=\frac{C_{2}V_{2}}{(C_{0}-C_{e})V_{1}}\times 100\%$$

In the above equation: C_0_ is the concentration of AATP in the sample solution (mg/mL); C_e_ is the concentration of AATP in the adsorption solution (mg/mL); C2 is the concentration of AATP in the desorption solution (mg/mL); V1 is the volume of the AATP adsorption solution (mL); V2 is the volume of the AATP desorption solution; m is the weight of the resin (g).

#### 2.3.2. Dynamic Sorption and Desorption

Dynamic leakage curves were studied during adsorption, and the effect of sample concentrations (0.4, 0.6, 0.8, 1.0, and 1.2 mg/mL) on the adsorption of AATP was determined. In addition, the effect of eluent ethanol volume fractions (50, 60, 70, 80, and 90%) on the elution capacity of AATP during desorption was investigated, and dynamic elution curves were plotted. The effect of upsampling and elution stream rates (1.0, 2.0, 3.0, and 4.0 mL/min) during dynamic sorption and desorption on the purification of AATP was investigated. When one of the factors was raised from the minimum to the maximum change, the other five factors were kept constant.

### 2.4. LC-MS

#### 2.4.1. Sample Preparation

In total, 25 mg of the AATP sample was weighed; 500 μL of methanol–acetonitrile–water = 2:2:1 (*V*/*V*/*V*) was added, ground, and sonicated, and this was repeated three times. The sample should then be left at −40 °C for one hour and centrifuged for fifteen minutes at 12,000 rpm and 4 °C (RCF = 13800× *g*, R = 8). Following centrifugation, the supernatant was filtered through a 0.22 µm membrane and cryopreserved at −80 °C in sterile containers for subsequent assessment.

#### 2.4.2. Sample Detection

The target chemicals were separated chromatographically using Vanquish ultra-performance liquid chromatography (UPLC) under the following chromatographic conditions. The chromatography’s A-phase was 0.01% acetic acid in water, and the B-phase was isopropanol–acetonitrile (1:1, *v*/*v*). Mass spectrometry analysis was performed on an Orbitrap Exploris 120 mass spectrometer with the following mass spectrometry conditions: sheath gas flow rate: 50 Arb; auxiliary gas flow rate: 15 Arb; capillary temperature: 320 °C; full ms resolution: 60,000; ms/ms resolution: 15,000; collision energy: SNCE 20/30/40; beam voltage: 3.8 kV (positive) or −3.4 kV (negative).

### 2.5. Cyberpharmacology and Molecular Docking

#### 2.5.1. Collection of Active Ingredient Targets of AATP

The relevant terpenoids contained in AATP were screened by literature mining and combined with preliminary LC-MS results. The standard 3D molecular structure and Smiles ID of AATP were downloaded from the PubChem database. The structures were entered into the SwissADME screen to derive suitable compounds. The compounds were then screened for relevant targets using Swiss Target Prediction (https://www.swisstargetprediction.ch/) and SEA Search Server (https://sea.bkslab.org/).

#### 2.5.2. Determine the Target

The keyword “atherosclerosis” was searched in four databases—GeneCards (https://www.genecards.org/), PharmaGKB (https://www.pharmgkb.org/), DrugBank (https://go.drugbank.com/), and Disgenet (https://www.pharmgkb.org/)—to identify known targets for atherosclerotic disease. The resulting data was plotted in a Venn map to reflect the number of targets associated with atherosclerosis. Following the removal of duplicate targets, a Venn diagram was constructed to visualize the intersection between AATP targets and atherosclerosis-related targets. The repeat target is the potential therapeutic atherosclerotic goal of AATP.

#### 2.5.3. Building the AATP PPI Network in Opposition

Protein–protein interaction (PPI) refers to the physical and functional interactions between proteins, which play crucial roles in genetic networks and biochemical pathways [42]. The target profiles of bioactive terpenoids from *A. absinthium* were intersected with known atherosclerosis targets. Subsequently, the authors utilized anti-atherosclerotic targets derived from the STRING database (https://string-db.org/) in order to build protein interaction networks. The minimum interaction score of ≥0.99 was selected, and the default parameters were employed for all other settings. The final step was to construct a protein–protein interaction network. The protein–protein PPI network was constructed using Cytoscape 3.7.2.

#### 2.5.4. Associated Signaling Networks Revealed by KEGG Pathway Enrichment Analysis

Gene ontology (GO) enrichment analysis and Kyoto Encyclopedia of Genomes (KEGG) signaling pathway enrichment analysis were performed using the Metascape database to annotate gene function (https://metascape.org/). The major objective of this research was to look at the antagonistic effects of AATP. KEGG advanced bubble plots were created using an internet program (https://www.bioinformatics.com.cn/), and pathways of interest at *p* < 0.05 were chosen for investigation and presentation.

#### 2.5.5. Molecular Docking

The total terpenoids of Artemisia annua and their related core targets JAK2 (PBD ID: 2B7A) and STAT3 (PBD ID: 6TLC) were subjected to molecular docking analysis to further validate the binding between the active components and the targets and to explore the potential target sites for the treatment of AS by the total terpenoids of Artemisia annua. The structural information of the active components of Artemisia annua’s total terpenoids was obtained from the PubChem database, and then, AutoDock 1.5.6 was used to determine the ligand’s rotatable bonds, etc. The 3D structures of the core targets were obtained from the PDB database, preprocessed, and then docked with the active components using AutoDock software. The docking results with binding energies meeting the requirements were visualized using PyMOL 2.5.2 software.

### 2.6. Study on the Anti-Atherosclerotic Effect of AATP

#### 2.6.1. Establishment and Administration of Animal Models

Xinjiang Medical University Laboratory Animal Center purchased the SD Rats. All experimental animals were maintained under controlled environmental conditions (22 ± 1°C, 55 ± 5% relative humidity) with a 12 h light/dark cycle. After 7 days of acclimatization, they were split into a control group and an atherosclerosis model group. For 16 weeks, the model group was fed a diet heavy in fat. VD3 (600,000 IU/kg) was injected intraperitoneally before modeling, and 100,000 IU/kg was injected 2, 4, 6, 8, and 10 weeks after modeling. The control group received normal food and water. At week 12 of modeling, animals were rectally divided into control, model, positive control (simvastatin, 5 mg/kg/d), and AATP (100 and 200 mg/kg) groups. The drug was delivered by gavage for 4 weeks. Rat body weights were measured weekly. The Animal Ethics Committee of Xinjiang University approved all animal experiments (approval number: XJUAE-2023-019).

#### 2.6.2. Serum Biochemical Indicator Tests

Centrifuge blood at 1000× *g* for 1800 s. The upper blood layer was then immediately shifted to a new test tube. Triglycerides (TGs), total cholesterol (TC), high-density-lipoprotein cholesterol (HDL-C), and low-density-lipoprotein cholesterol (LDL-C) were measured according to the reagent instructions.

#### 2.6.3. Histopathological Observation

After being submerged in a 10% formaldehyde solution, the liver and aorta were gradually dehydrated using a series of graded ethanol solutions and then cut into pieces that were 3–5 μM thick. Light microscopy was used to evaluate the pathology of rat liver and aorta after hematoxylin and eosin staining.

#### 2.6.4. Cell Culture

RAW 264.7 cells are murine macrophage lines, obtained from the Cell Bank of the Chinese Academy of Sciences. RAW 264.7 cells were cultured in Dulbecco’s modified Eagle’s medium (DMEM) containing 10% fetal bovine serum (Gibco, Waltham, MA, USA) in a humidified incubator at 37 °C and 5% CO_2_ (Thermo Fisher Scientific, Waltham, MA, USA).

#### 2.6.5. Cell Viability Assay

After the cells were fully grown, they were treated with different concentrations of AATP (5, 10, 20, 30, 40, and 50 μg/mL) and 100 μL of complete medium as a normal reference. After 24 h of incubation, the influence of drugs on cell viability was tested by the MTT method.

#### 2.6.6. Oil Red O Staining

Then, 96-well plates were inoculated with 5 × 10^4^ RAW264.7 cells per well, which were subsequently randomly assigned to the control, model, and culture groups. (80 µg/mL OX-LDL), a positive control group (5 µM simvastatin), and an AATP group (5, 10, and 20 µg/mL). After dissolving Oil Red O in isopropanol, the absorbance was measured at 550 nm.

#### 2.6.7. Inflammatory Factor Assay

The inflammatory factors in the serum of the rats, including TNF-α and IL-6, were tested using ELISA detection kits. In a 24-well plate, inoculate RAW264.7 cells and incubate for 24 h. Following randomization, the model group only received the 100 ng/mL lipopolysaccharide (LPS) treatment, whereas the control group was provided the entire medium. The drug group used 100 ng/mL LPS and different amounts of AATP (5, 10, and 20 μg/mL). After 24 h of cell incubation, the supernatant from each group was collected. The levels of IL-6 and TNF-α secretion were quantified by ELISA methods.

#### 2.6.8. ROS Detection

RAW264.7 cells were seeded in 6-well plates at a volume of 2 mL (2 × 10^5^/mL) per well and incubated for 24 h. RAW264.7 cells were pretreated with LPS and AATP for 24 h. Subsequently, cells were cultured with 10 μM DCFH-DA for 30 min. Finally, DCF fluorescence intensity was detected by flow cytometry in the FITC pathway. The fluorescence intensity of DCFH-DA was observed under an inverted fluorescence microscope.

#### 2.6.9. Flow Cytometry Analysis

Examine the representation of CD86 on AATP-treated RAW264.7 cells by flow cytometry. The treated RAW264.7 cells were taken and incubated for 15 min with the APC-coupled anti-mouse CD86 antibody, as per instructions. Quadrant segmentation analysis yielded the mean fluorescent signal intensity.

### 2.7. Statistical Analysis

Statistical data analysis was performed using GraphPad Prism 8.0 software. All experiments included at least three independent replicates. The results are expressed as the mean ± standard deviation. Comparisons between multiple groups were performed using one-way ANOVA combined with Tukey’s multiple-comparison test. The specific sample size (*n* value) and statistical significance (*p* value) are noted in the corresponding figure captions, with *p* < 0.05 indicating statistical significance. Data visualization was performed using GraphPad Prism software.

## 3. Results

### 3.1. Single-Factor Experimental Results of AATP Extraction

#### Results of Single-Factor Experiments with Different Ethanol Concentrations

**Figure 1:**
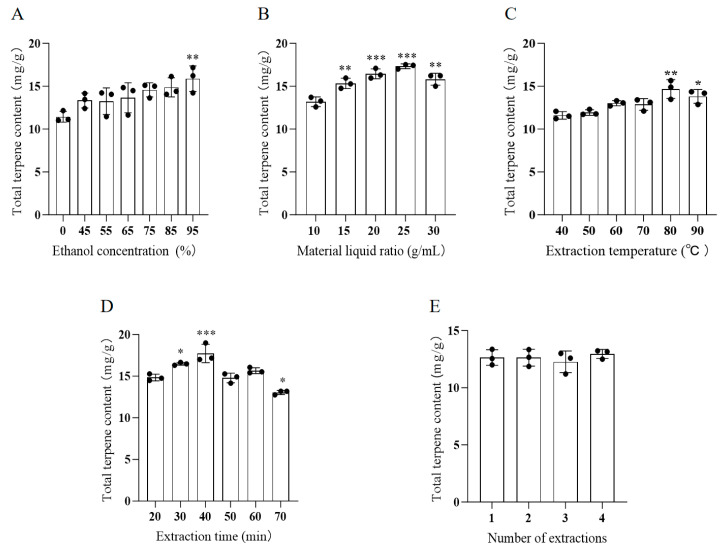
The effects of (**A**) ethanol concentration, (**B**) material–liquid ratio, (**C**) extraction temperature, (**D**) extraction time, and (**E**) number of extractions on the total terpene content were investigated by a single-factor experiment. Compared with the first group:*** *p* < 0.001 ** *p* < 0.01 and * *p* < 0.05.

Figure 1A shows that the total terpenoid content of wormwood increases with increasing ethanol concentration. When the ethanol concentration reaches 95%, the total terpenoid content reaches its maximum value. It may be that as the ethanol concentration increased, the terpenoids were better leached, and hence, the content increased (Figure 1A). The total terpenoid content of wormwood shows an initial increase followed by a decrease as the ratio of sample mass to extraction solvent volume increases. The total amount of terpenoids was maximized at a solid-to-liquid ratio of 1:25 g/mL. However, the amount of AATP decreased when the feed–liquid ratio exceeded 1:25. The observed effect could originate from the low material–liquid ratio, which reduces the concentration gradient between phases. This diminished gradient hinders solute diffusion in the solvent, thereby impeding terpenoid dissolution (Figure 1B). The total terpene content of absinthia increased slowly with increasing temperatures when the extraction temperature was between 40 and 80 °C. This may be due to the leaching of terpenoids by the gradual increase in temperature. The highest total terpene levels were observed at an abstraction temperature of 80 °C, while the total terpene levels decreased when the temperature exceeded 80 °C. The extracts exhibited significantly elevated terpene concentrations compared to the raw material. Maximum terpenoid yields were achieved at 80 °C, with a marked decline observed beyond this threshold temperature. This may be due to the change in the structure of terpenoids under high-temperature conditions (Figure 1C). Given the brief extraction period at the outset, the terpenoids were not fully extracted. The extracted content of AATP reached its maximum value at 40 min, beyond which the further extension of the extraction time may have resulted in competition with other impurities, leading to a reduction in the extracted content (Figure 1D). As the number of extractions increased, the AATP content tended towards stability. This may be due to the fact that after a certain number of extractions, the amount of AATP reaches equilibrium. In consideration of the actual production process and the rational and efficient use of resources, the quantity of extractions is determined to be one (Figure 1E).

That is, the one-way experiment yielded the following conditions for AATP extraction: an extraction solvent of 95% ethanol, a material-to-liquid ratio of 1:25 g/mL, an extraction temperature of 80 °C, an extraction time of 40 min, and a single extraction.

### 3.2. Analysis of Extraction Parameters Using Response Surface Method

#### 3.2.1. Response Surface Regression Model and Profiling of Variance

Response surfaces are an integrated statistical testing technique for addressing the consequences of multiple elements of a structure or system. In other words, they are used to investigate the transformational relationship between the inputs (variable values) and the outputs (responses) of a framework or system [43]. The data indicate that the material-to-liquid ratio, extraction temperature, and extraction time have a marked influence on the results of the extraction. The response surface analysis scheme and results are presented in Table 1 and Table 2. After multiple regression analysis, the following binary multiple recurrence model was derived:

Y = −168.23 + 3.93A + 2.8024B + 1.16C + 0.0028AB + 0.012AC + 0.00054BC − 0.092A2 − 0.017B2 − 0.018C2.

The ANOVA of the response surface model in Table 3 indicates that the regression model is highly notable (*p* < 0.001) and that the difference is statistically significant. The mismatch term *p* = 0.4395 > 0.05 indicates that the mismatch is not significant (** *p* < 0.01). This suggests that the model fits well with the experiment and that the experiment produces fewer errors. The R2 value of 0.9886 indicated that 98.86% of the variation in the total terpenoids content of absinthe was attributable to the selected experimental factors. Table 3 shows the test results obtained under different combinations of experimental conditions. The regression equations accurately reflect the link between the experimental parameters and the response values, thus helping to predict and analyze the parameters related to the AATP extraction process. The modified coefficient of determination, R^2^ = 0.9739, indicated that the model could account for 97.39% of the observed change in the response values; The ANOVA data from the fitted regression equation proved that terms B, AC, A2, B2 and C2 in the first term of the equation had a significant effect on the extracted content of AATP. Conversely, the remaining terms were not found to be marked, indicating that the relationship between the experimental factors and the AATP content was not a straightforward linear relationship. As illustrated in the table, the F-values for the individual factors A, B, and C were 4.73, 5.76, and 0.76, respectively. This indicates that the degree of influence of these three factors on the total terpene content was B > A > C.

#### 3.2.2. Response Surface Interaction Analysis

**Figure 2:**
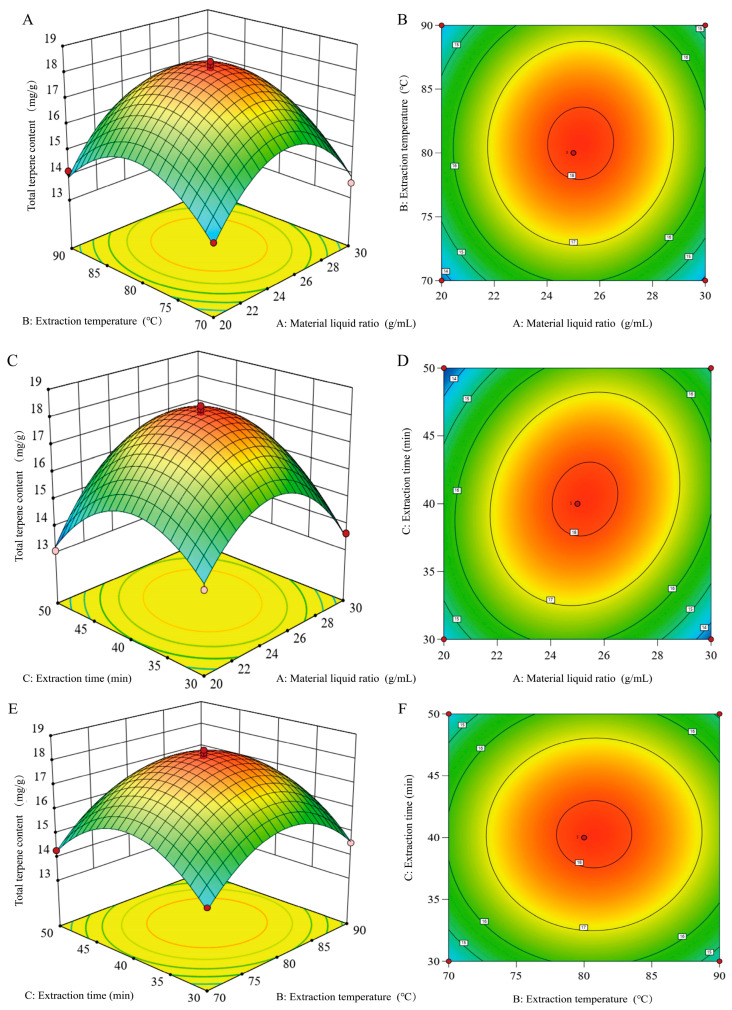
Response surface plots (**A**–**C**) and contour plots (**D**–**F**) showing the effects of different extraction parameters on the yield of total terpenoids. (**A**,**D**) Material–liquid ratio and extraction temperature. (**C**, **D**) Material–liquid ratio and extraction time. (**E**, **F**) Extraction temperature and extraction time.

The test results were used to plot response surfaces and contour lines, as illustrated (Figure 2), to assess the effects of every extraction condition on the sum terpene content and the interactions between the factors. Dynamic associations between factors can be resolved by response surface slope analysis. If the response surface’s slope is greater, the total terpene content of absinthe is more impacted by the combination of the two elements. Conversely, if the effect is smaller, the shape of the dense contour is elliptical, suggesting that the way the two interact has a noteworthy impact on the response value. In contrast, the circular structure of the sparse profile suggests that the effect of the interaction is not significant [43]. The analysis revealed that the response surface slope for the A and C interaction was the highest, and the ellipticity of its contour plot was the greatest, indicating that the most noticeable impact on the total terpene content was caused by the interplay between the ratio of the material to the liquid and the duration of extraction. The material–liquid ratio and extraction temperature, together with the temperature and duration of extraction, exhibited minimal influence, and the ellipticity of the contour plots was not conspicuous, suggesting that the interaction was not statistically significant.

#### 3.2.3. Determination and Verification of Optimal Conditions for Optimization

The model analysis indicated that ideal circumstances for the extraction of total terpenoids from absinthe were as follows: a material-to-liquid ratio of 25.30 g/mL, an extraction heat level of 80.77 °C, and a recovery time of 40.36 min. The experimental conditions were adjusted as follows: The material–liquid ratio was 25 g/mL, the extraction temperature was 80 °C, and the extraction time was 40 min. The total terpene content measured was 18.00 ± 0.28 mg/g, which is not much different from the theoretical value of 18.15 mg/g. The total terpene content of 18.00 ± 0.28 mg/g is not very different from the theoretical value.

### 3.3. Purification Results of Macroporous Resins

#### 3.3.1. Selection of Macroporous Resins

**Figure 3:**
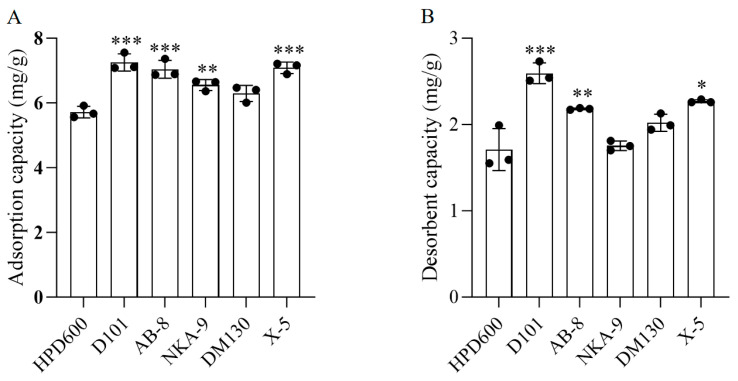
Static adsorption (**A**) and desorption (**B**) effects of different types of macroporous resins. Compared with the HPD600 group: *** *p* < 0.001, ** *p* < 0.01, and * *p* < 0.1.

The chemical structure, polarity, specific surface area, and hole size of the sorbent material are important factors affecting the adsorption and desorption of the resin [44,45]. Through balanced adsorption and subsequent desorption studies, six types of macroporous resins were systematically screened to select a resin for the purification of AATP. The outcomes, as displayed in Table 4 and Figure 3, revealed that the D101 resin exhibited superior adsorption and desorption effects on AATP compared with the other five resins. Consequently, the D101 resin was selected for use in the purification of AATP in this experiment according to its superior properties of adsorption and desorption.

#### 3.3.2. Results of Dynamic Absorption Experiments

**Figure 4:**
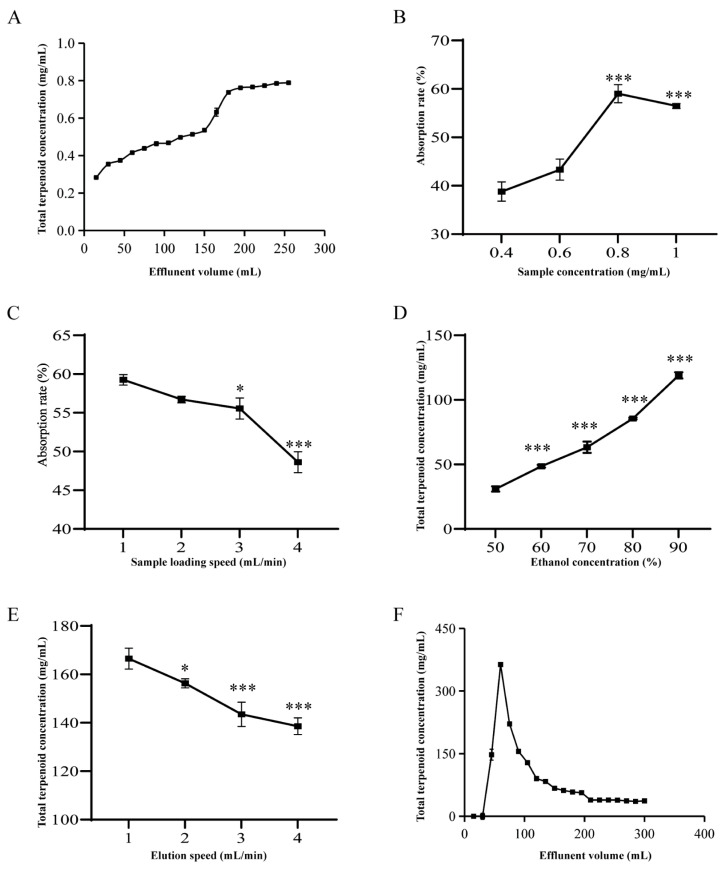
Factors affecting the adsorption and desorption performance of D101 resin. (**A**) Leakage curves. (**B**) Effect of sample concentration values on adsorption capacity. Compared with the first group: *** *p* < 0.001. (**C**) Effect of sample flow rate on adsorption capacity. Compared with the first group: *** *p* < 0.001 and * *p* < 0.1. (**D**) Effect of eluent ethanol concentration on desorption capacity. Compared with the first group: *** *p* < 0.001. (**E**) Effect of elution flow rate on resolving capacity. Compared with the first group: *** *p* < 0.001 and * *p* < 0.1. (**F**) Elution curve.

The sorption of macroporous resins on the target is subject to alterations when different dynamic adsorption and desorption conditions are employed, which may have implications for the interpretation of experimental results. The total terpenoid concentration in the effluent demonstrated a gradual increase with the expansion of the sample volume, and a considerable quantity of terpenoids commenced flow at 150 mL. When the volume reaches 200 mL, the total terpene concentration in the effluent reaches a stable equilibrium, suggesting that the resin has reached its sorption point of saturation. This phenomenon may be due to the dynamic sorption process of macroporous resins, whereby the ability to adsorb terpenoids diminishes after reaching saturation. As a consequence, the unsuccessful adsorption of terpenoids occurs, resulting in their leakage from the resin. This results in the stabilization of the terpenoid concentrations in the effluent at 200 mL (Figure 4A). When the sample’s concentration rises, the resin does not saturate, allowing for the adsorption of terpenoids and increasing the rate of adsorption. However, when the concentration of such a sample is very large, the number of impurities in the solution also increases. The impurities and the target compounds then compete for the limited active sites of the resin, causing a decrease in adsorption capacity. Furthermore, when the concentration was excessive, it caused the resin to become clogged, which in turn affected the adsorption effect [46]. The resin achieved peak sorption efficiency at a sample concentration of 0.8 μg/mL (Figure 4B). The experimental results show that both adsorption and desorption decrease with increasing flow rates. The flow rate was excessive, which caused a decrease in the sorption affinity of the macroporous resin for the target species. Moreover, the AATP did not achieve sufficient contact with the resin, resulting in incomplete adsorption or elution. Given the requisite productivity and time constraints, the stream rates of 1 mL/min and 2 mL/min were thus identified as optimal for upsampling and elution, respectively (Figure 4C, E). The highest total terpenoid concentration was observed when ethanol reached a concentration of 90% during resin elution. This effect could be explained by AATP’s increased solubility in high ethanol concentrations (Figure 4D). The total terpenoids were predominantly eluted from the resin once the volume of eluent reached 210 mL (Figure 4F).

In summary, the preferred process data for the purification of AATP by D101 macroporous resin were as follows: sample consistency of 0.8 mg/mL, sample volume of 150 mL, rate of effusion of 1.0 mL/min, eluent of 90% ethyl alcohol, rate of effusion of 2.0 mL/min, and volume of effusion of 210 mL. Before and after purification, the purity of AATP increased from 20.85% ± 0.94% to 52.21% ± 0.75% and that of AATP was increased from 20.85% ± 0.94% to 52.21% ± 0.75%. Before and after purification, the purity of AATP increased from 20.85% ± 0.94% to 52.21% ± 0.75%.

### 3.4. Outcomes of Cyberpharmacology

#### 3.4.1. AATP and Potential Targets in Atherosclerosis

**Figure 5:**
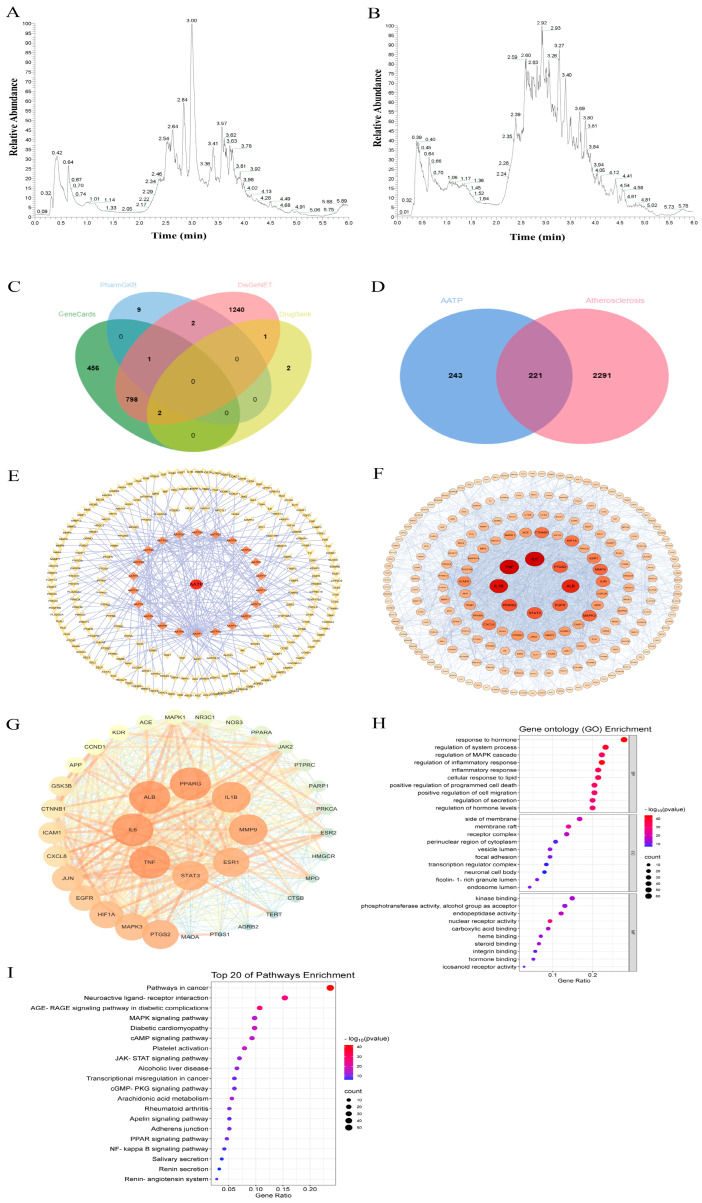
LC-MS (**A**) positive and (**B**) negative ion chromatograms of AATP. (**C**) Venn diagram of disease-associated targets. (**D**) Venn diagram of active ingredient-disease-associated targets. (**E**) Network diagram of the active ingredient’s potential targets. (**F**) PPI network of intersecting targets of AS and AATP. (**G**) Key targets of AATP for AS based on the CytoNCA analysis. (**H**) GO enrichment analysis. (**I**) KEGG pathway enrichment analysis.

The constituents of AATP were characterized by LC-MS, and the chromatograms were given in positive and negative ion modes (Figure 5A, B). A total of 22 active ingredients of AATP were identified through database screening as potential treatments for AS. A search of the GeneCards, DisGeNET, PharmGKB, and DrugBank databases identified 3320 targets associated with atherosclerosis. The distribution of the 3319 targets according to their compositional relevance was as follows: 1257 in GeneCards, 2044 in DisGeNET, 13 in PharmGkb, 13 in DrugBank, and 5 in DrugBank (Figure 5C). Following the elimination of duplicate values, a total of 2251 disease targets were identified. Subsequently, Venn diagrams were constructed to identify 221 target genes that were common to both the AATP-effective ingredient targets and the atherosclerosis targets (Figure 5D). The 221 targets were deemed to be prospective candidates for AATP-mediated anti-atherosclerosis. Subsequently, the active-ingredient-targeted network of protein–protein interactions was built using Cytoscape 3.7.2 (Figure 5E), which is accessible for further investigation. The potential target network is depicted (Figure 5F), which suggests a close association between the targets. Thirty-seven key targets were screened according to the degree value, median centrality (BC), and neighboring centralities (CC) (Figure 5G), including STAT3, JAK2, TNF, IL-6, PPARG, and IL-1β.

#### 3.4.2. GO and KEGG Pathway Enrichment Analysis

The Metascape database was utilized for GO and KEGG enrichment studies of relevant AATPs against atherosclerotic targets. The GO functions mainly included kinase binding, steroid binding, and the regulation of cellular inflammatory responses (Figure 5H), while the KEGG-enriched pathways primarily included the JAK/STAT signaling pathway, the PPAR signaling pathway, the platelet activation pathway, and the cAMP signaling pathway, among others (Figure 5I). This suggests that AATP may inhibit the development of atherosclerosis by modulating many biological processes and the JAK/STAT signaling pathway.

#### 3.4.3. Molecular Docking Analysis

**Figure 6:**
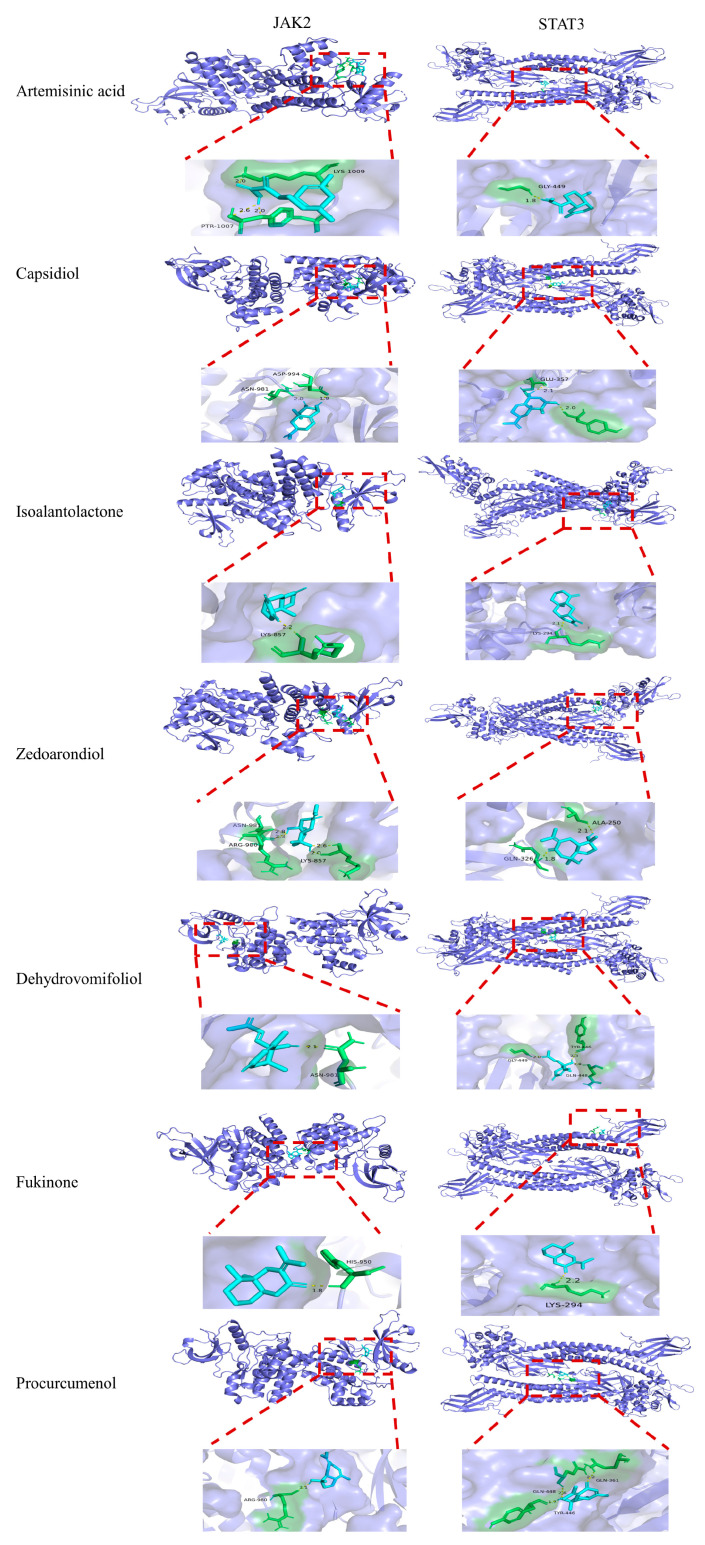
The three-dimensional conformation of the AATP active ingredient (artemisinic acid, calcidiol, isoalantolactone, zedoarondiol, dehydrovomifoliol, quinone, and procurcumenol) docked to JAK2 and STAT3 target protein molecules.

Based on the target proteins (STAT3 and JAK2) predicted by network pharmacology analysis and the related inflammatory signaling pathway JAK/STAT, molecular docking was performed between JAK2 and STAT3, and the active components (Artemisinic acid, Capsidiol, Isoalantolactone, Zedoarondiol, Dehydrovomifoliol, Fukinone, and Procurcumenol) were subjected to molecular docking (Figure 6). The binding efficiency of each component with the target protein was indicated by the magnitude of the binding energy. A binding energy below −4.25 kcal/mol typically indicates the presence of binding activity between the receptor and ligand, while a binding energy below −5.00 kcal/mol corresponds to a stable binding conformation. The results are shown in Table 5. The binding free energies of the active components with the target proteins were all below −5.00 kcal/mol, indicating spontaneous and stable interactions. Therefore, AATP can effectively and stably bind to the key target sites predicted by network pharmacology.

**Table 5:** Molecular docking binding energy (in kcal/mol).

Compound	JAK2	STAT3
Artemisinic acid	−5.28	−5.80
Capsidiol	−5.94	−5.64
Isoalantolactone	−5.84	−6.12
Zedoarondiol	−5.59	−5.80
Dehydrovomifoliol	−5.03	−5.75
Fukinone	−5.67	−6.22
Procurcumenol	−6.03	−6.40

#### 3.4.4. Effects of AATP on Body Weight and Organ Indexes in Atherosclerotic Rats

**Figure 7:**
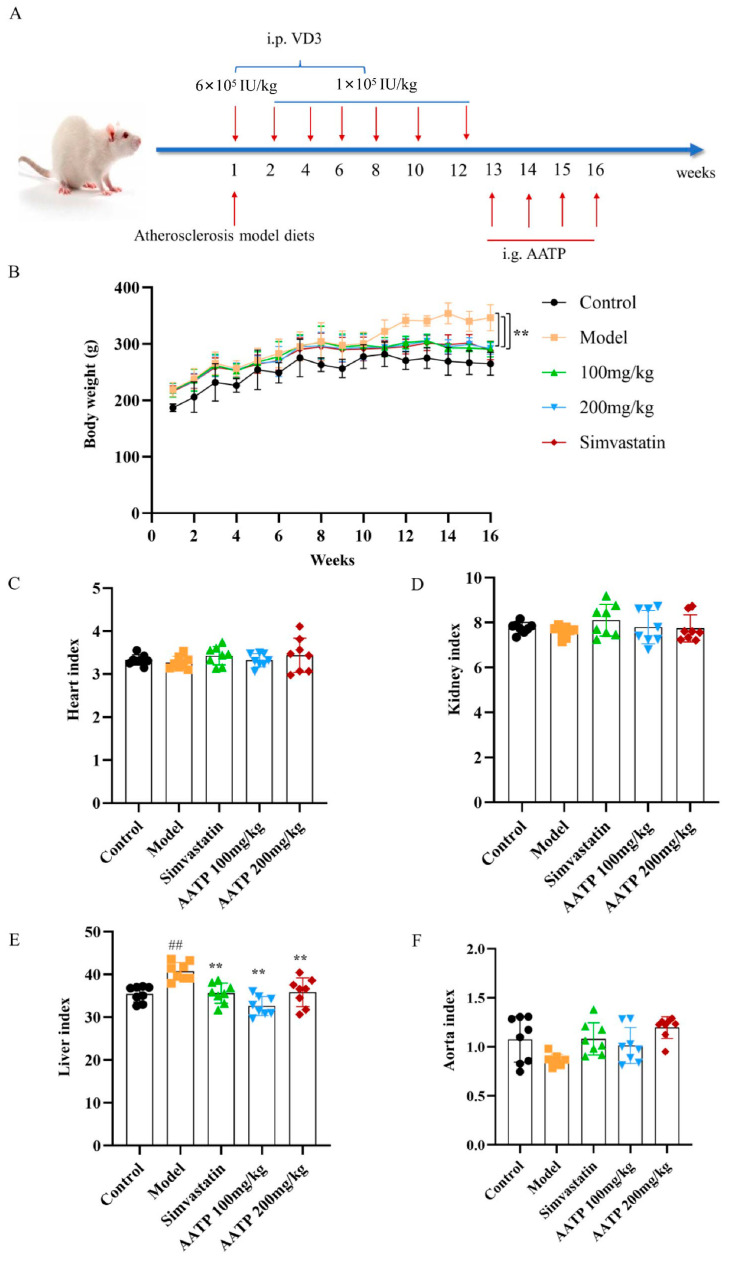
Effect of AATP on body weight and organ indices in AS rats (mean ± SD, *n* = 8). (**A**) Modeling AS with high-fat feeds combined with vitamin D3. (**B**) Changes in body weight of rats. Changes in (**C**) heart, (**D**) kidney, (**E**) liver, and (**F**) aortic weight indices. Compared with the control group: ## *p* < 0.01; compared with the model group: ** *p* < 0.01.

An animal model of atherosclerosis was created by administering a high-fat diet and injecting VD3. Following AATP intervention, a gradual increase in body weight, followed by a gradual decrease in body weight in rats eating fatty foods (Figure 7A), was observed. In Figure 7B, the post-treatment weight was significantly different from that of the model group (*p* < 0.01). A significant increase in liver index was noted within the model group of rats in comparison to the control group (*p* < 0.01) in the organ index analysis. However, compared with model rats, liver indicators were significantly reduced after AATP treatment (*p* < 0.01), while the indicators of other organs remained basically unchanged and were not statistically significant (Figure 7B–D, F). This suggests that AATP does not produce marked toxic actions on the organs of AS rats and attenuates the abnormal proliferation and hypertrophy of the liver in AS rats.

#### 3.4.5. AATP Attenuates Aortic and Hepatic Lesions in AS Rats

**Figure 8:**
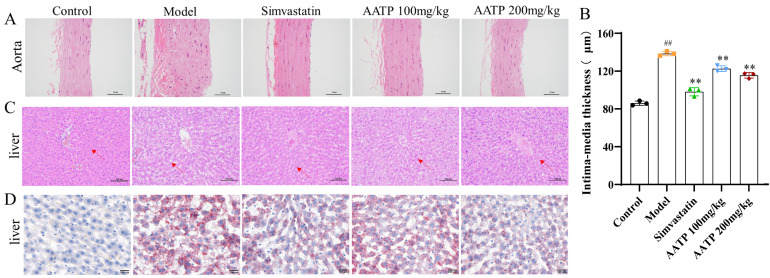
(**A**) HE staining of the aorta. (**B**) Aortic intima-media thickness. (mean ± SD, *n* = 3). (**C**) HE staining of the liver. (**D**) Oil red O staining of the liver. Compared with the control group: ## *p* < 0.01; compared with the model group: ** *p* < 0.01.

Tissue staining was performed on the rats to visualize the lesions. The model’s aortic wall was noticeably thicker than that of the control group. AS rats (*p* < 0.01) and most of the cells in the wall showed irregular arrangement. Compared with the model group, a thinner aortic wall and more aligned cells were observed visually in the AATP and simvastatin groups (*p* < 0.01) (Figure 8A, B). Moreover, the livers of the model group exhibited disorganization and an increased number of fat vacuoles in contrast to the control group. However, following gavage therapy using AATP and simvastatin, cells in the liver are organized, and the fat vacuoles were significantly reduced (Figure 8C). Furthermore, in comparison to the control group, the livers of the model group exhibited a notable accumulation of fat. In contrast, the administration of AATP via gavage resulted in a reduction in the accumulation of lipids in the liver, accompanied by a notable decline in the number of lipid droplets (Figure 8D). These findings indicate that AATP may possess the potential to mitigate the thickening of the aortic wall in rat AS, suppress the formation of substantial quantities of fat vacuoles in the liver, and attenuate the accumulation of lipids. It may also help inhibit the progression of aortic and liver lesions.

#### 3.4.6. AATP Improves Lipid Levels, Liver Function, and Inflammatory Factors

**Figure 9:**
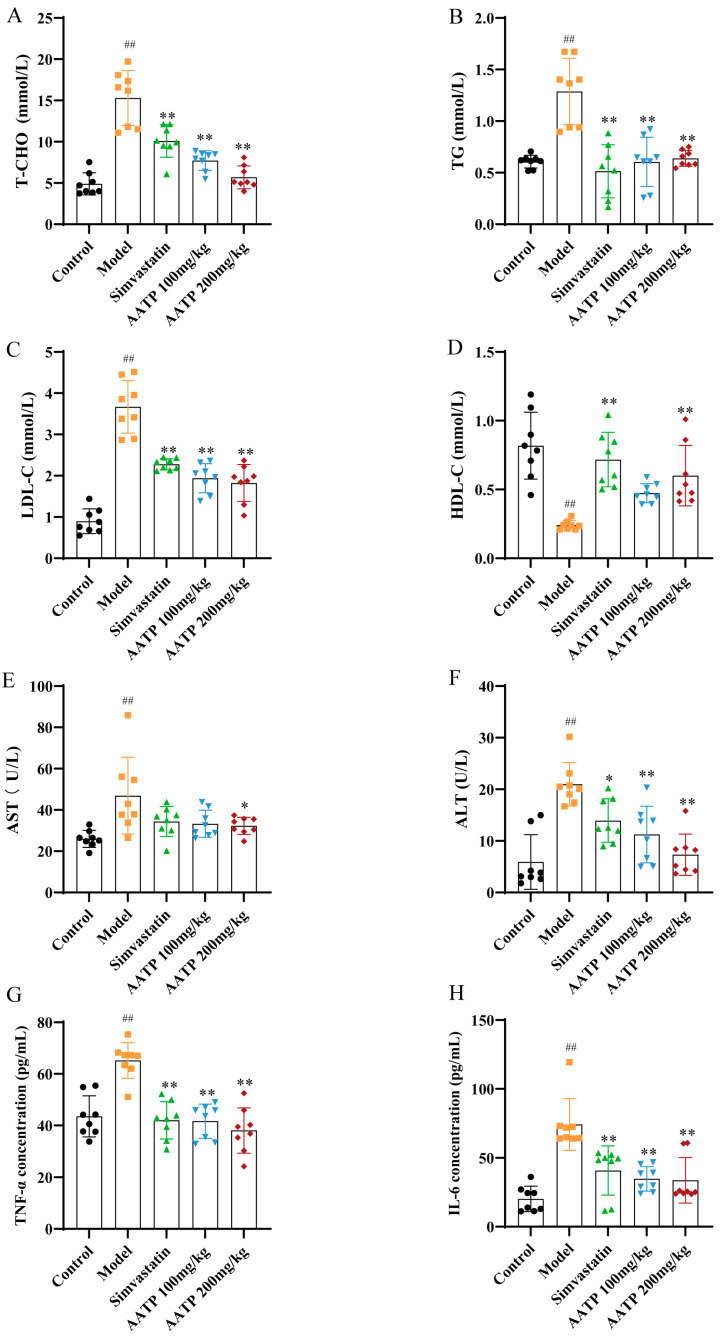
AATP effects on rat lipid, liver, and cytokine levels (mean ± SD, *n* = 8). Serum concentrations of (**A**) TC, (**B**) TG, (**C**) LDL-C, (**D**) HDL-D, (**E**) AST, (**F**) ALT, (**G**) TNF-α, and (**H**) IL-6. Compared with the control group: ## *p* < 0.01; compared with the model group: * *p* < 0.05; ** *p* < 0.01.

Compared with the control group, the serum levels of TC, TG, and LDL-C were found to be significantly elevated in the model group of rats, even though HDL-C was significantly decreased (*p* < 0.01). In contrast, all other indices were observed to be reduced in both the Stimavastin group and the AATP group, except for the increase in HDL-C (*p* < 0.01) (Figure 9A–D). A subsequent assessment of serum liver function indices, aspartate aminotransferase (AST), and alanine aminotransferase (ALT) demonstrated that AATP was effective in decreasing the blood levels of AST and ALT in AS rats, indicating that it improves impaired hepatic function due to long-term hepatic steatosis (Figure 9E,F). Given the well-documented role of inflammation in promoting atherosclerotic plaque formation, an investigation was also conducted into the effect of AATP on inflammatory factors. This study’s findings demonstrated that serum TNF-α and IL-6 levels were considerably raised in AS rats compared with the control group (*p* < 0.01). Significantly lower levels of TNF-α and IL-6 were found after AATP treatment compared with the model group. In conclusion, AATP proved effective in improving lipid levels, liver function, and inflammatory cytokine levels in AS rats.

#### 3.4.7. Effect of AATP on the Viability of RAW264.7 Cells

**Figure 10:**
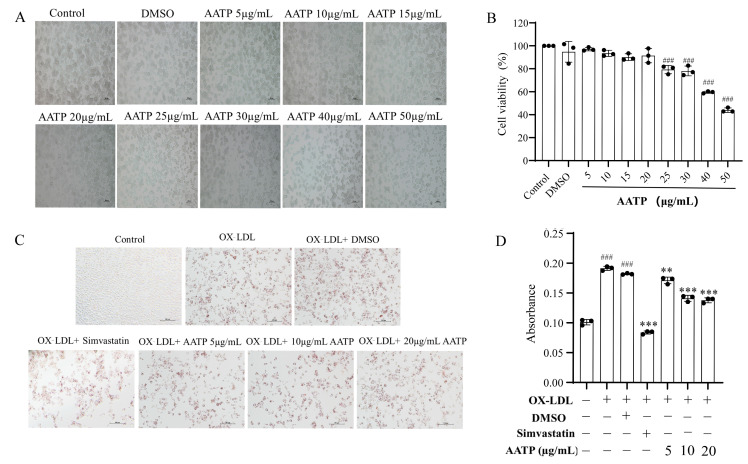
Impact of AATP on (**A**) viability and (**B**) morphology of RAW264.7 cells. (**C**) Oil red O staining of RAW264.7 cells. (**D**) Quantification of oil red O content at 550 nm. Compared with the control group: ### *p* < 0.001; compared with the model group: ** *p* < 0.01 and *** *p* < 0.001.

Compared with the control group, no notable cytotoxic impact on RAW264.7 cells was observed when the AATP concentration remained below 20 μg/mL (*p* > 0.05) (Figure 10A). Furthermore, microscopic observation revealed a notable reduction in cell number at concentrations exceeding 20 μg/mL (Figure 10B). Accordingly, the AATP at concentrations of 5, 10, and 20 μg/mL was selected for subsequent experimentation.

#### 3.4.8. AATP Inhibits Ox-LDL-Induced Foam Cell Formation

Macrophages can take up OX-LDL to form foam cells, which ultimately leads to plaque destabilization and rupture [47]. To further investigate the effect of AATP on plaque vulnerability, an OX-LDL treatment was applied to create a macrophage-derived foam cell model. No notable formation of lipid droplets was observed in the normal control cells, whereas a considerable increase in lipid droplets was evident within the model cells that were contrasted with the controls (the concentration of DMSO is the concentration of DMSO in the highest drug dose group) (*p* < 0.001). Following intervention with AATP at levels of 5, 10, and 20 μg/mL, a pronounced reduction in intracellular lipid droplets was observed (*p* < 0.001) (Figure 10C,D). The findings suggest that AATP may be able to reduce intracellular lipid accumulation and impede OX-LDL-induced foaminess in RAW 264.7 macrophages. This suggests that AATP may be involved in the prevention of atherosclerosis.

#### 3.4.9. AATP Suppresses Pro-Inflammatory Mediator Synthesis in RAW264.7 Macrophages

**Figure 11:**
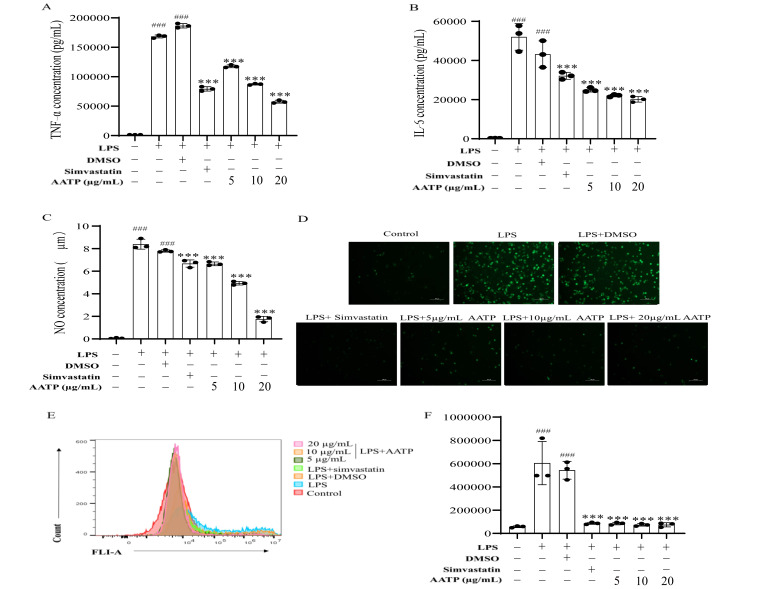
Cellular concentrations of (**A**) TNF-α, (**B**) IL-6, and (**C**) NO. (**D**) Inverted fluorescence microscopy to observe the effect of AATP on ROS. (**E**,**F**) Flow cytometry to detect ROS. Compared with the control group: ### *p* < 0.001; compared with the model group:*** *p* < 0.001.

Macrophages initiate the proinflammatory process by launching a variety of pro-arthritic mediators and contribute to the worsening of a number of inflammation-associated diseases [7,48]. AATP treatment resulted in significantly attenuated inflammatory responses relative to simvastatin, as evidenced by reduced cytokine secretion (*p* < 0.001) (Figure 11A–C). In addition, there is evidence that LPS stimulates the infiltration of inflammatory cells into lung tissue, resulting in the release of large amounts of reactive oxygen species. This, in turn, allows inflammatory cells to accumulate, promoting an inflammatory reaction that may affect atherosclerosis [49]. The administration of LPS resulted in a notable elevation in intracellular ROS production, whereas the administration of AATP was observed to exert a pronounced inhibitory effect on ROS generation (*p* < 0.001) (Figure 11D–F). Experimental data revealed that AATP exhibited significant anti-inflammatory activity by inhibiting the secretion of key inflammatory factors (e.g., IL-6, TNF-α).

#### 3.4.10. AATP Inhibits M1 Polarization in RAW264.7 Macrophages

**Figure 12:**
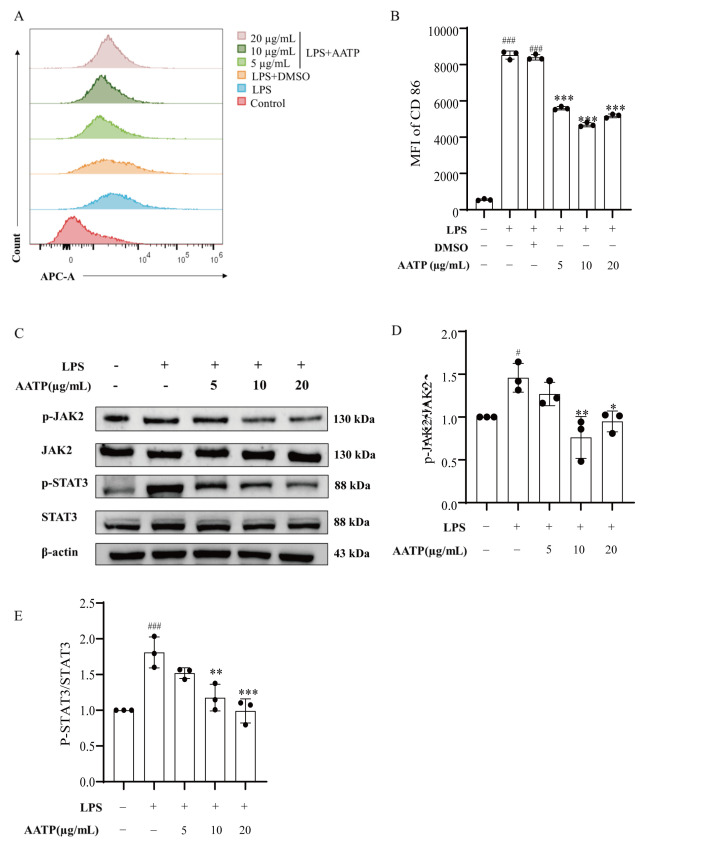
(**A**,**B**) Effect of AATP on CD86 expression on the surface of RAW264.7 cells using flow cytometry. (**C**–**E**) Effect of AATP on the expression of JAK2, p-JAK2, STAT3, and p-STAT3 proteins in RAW264.7 cells. Compared with the control group: # *p*< 0.05; ### *p* < 0.001; compared with the model group: * *p* < 0.05, ** *p* < 0.01, and *** *p* < 0.01.

Activated M1 macrophages secreted multiple proinflammatory cytokines, which promoted inflammatory progression and contributed to atherosclerosis development. To determine whether AATP alleviated atherosclerosis by inhibiting M1 macrophage polarization, CD86 expression (an M1 polarization marker) was quantified using flow cytometry. The results demonstrated significantly elevated CD86 expression in LPS-treated model groups versus controls (*p* < 0.001), whereas AATP treatment markedly reduced surface CD86 levels compared to model groups (Figure 12A, B). These findings indicated that AATP effectively suppressed LPS-induced M1 polarization in RAW264.7 macrophages.

#### 3.4.11. AATP Modulated JAK2/STAT3 Pathway

It is widely accepted that activation of the JAK/STAT signaling pathway is a hallmark of the inflammatory process. The LPS-induced cytokine cascade in macrophages initiated JAK-receptor complex formation and downstream STAT phosphorylation events [50]. Beyond that, the activated JAK/STAT signaling pathway has been proven to promote aberrant pro-inflammatory cytokines and to regulate macrophage polarization, eventually leading to inflammatory diseases [51]. Protein immunoblotting demonstrated that JAK2 and STAT3 phosphorylation was markedly elevated (*p* < 0.05) in LPS-induced RAW264.7 cells, whereas total absinthium terpenoids (10 and 20 μg/mL) exhibited a pronounced inhibitory effect on JAK2 and STAT3 phosphorylation (*p* < 0.05) (Figure 12C–E). Thus, AATP may inhibit the atherosclerotic process by regulating the JAK2/STAT3 signal transduction cascade.

## 4. Discussion

*Artemisia absinthium* L. plays a prominent role in traditional medicine. It is a therapeutic plant with important medicinal and economic values [52]. In recent years, extensive research has been conducted on the chemical composition of *A. absinthium* raw materials and the bioactivity of its extracts. These studies identified essential oils, bitter sesquiterpene lactones, flavonoids, other bitter compounds, and chamomile derivatives as the primary bioactive constituents [53]. Terpenoids, which are plentiful in medicinal plants, make up a category of natural active products with an extensive range of medicinal actions and pharmacological activity and a multitude of applications utilized to treat a variety of diseases. They exhibit considerable potential for further development and application. As a class of natural products, terpenoids exhibit structural diversity, comprising multiple subclasses. Each subclass of terpenoids possesses distinctive biological properties [26].

Multiple factors affected the extraction process, including the plant material’s particle size, solvent type, solvent-to-solid ratio, extraction duration, and processing temperature [54]. Attar et al. [55] reported that conventional Soxhlet extraction demonstrated superior efficiency in extracting tetracyclic triterpene cucurbitacin I from Cucurbita pepo compared to microwave-assisted and ultrasound-assisted extraction techniques. Response surface methodology (RSM), a statistical optimization approach, was widely applied in process optimization studies due to its capacity to expand the parameter space [56]. This method proved effective for optimizing, designing, and improving processes where output variations depended on multiple input variables [57]. The Box–Behnken design (BBD) can be fitted by a non-linear model to better reflect the objective reality of the process conditions, and at the same time, it can take into account the different influencing factors and evaluation indexes in the experimental design of the accuracy of the outcomes of the precision prediction of the optimization method based on the BBD. Compared with other methods, BBD optimization demonstrated the ability to reduce the total number of experiments required for process optimization. The optimized protocol exhibited simplicity, rationality, and stability, providing a methodological foundation for enhancing natural product extraction processes [58]. The authors use response surface optimization to analyze the factors affecting the response with a fitted model and find the optimal operating conditions. This approach saves time and is more operational.

First, a one-way experimental design was implemented to evaluate the effects of ethanol concentration, material-to-liquid ratio, extraction temperature, extraction duration, and extraction cycles on AATP yield. Subsequently, these parameters were further optimized using response surface optimization (RSD) to determine the optimal extraction conditions for AATP. Combining the results of single factor and BBD response surface design optimization, the optimal extraction process of total terpenoids from *A. absinthium* was obtained as follows: 95% ethanol as the extraction solvent, 1:25 g/mL as the material–liquid ratio, 80 °C as the extraction temperature, and 40 min as the extraction time. Additionally, there was only one extraction. Examining the impact of the feed-to-liquid ratio on total terpenoid extraction, the AATP content first increased and then decreased with an increase in the feed–liquid ratio. This might be because when the feed–liquid ratio is too low, there is little difference in concentration between the liquid and feed phases, which hinders the diffusion of substances in the solvent and is not conducive to the production of terpenoids in large quantities of the active substances being leached. As a result, the total amount of terpenoids is relatively small; as the material-to-liquid ratio steadily increases, the specific surface area of the molecules with solvent exposure increases. This process allows for the gradual dissolution of terpenoids, thereby increasing the total amount of terpenoids in the material. However, an excess of solvent can lead to a dilution effect, accompanied by a rise in the concentration of impurities in the extracted solution. Consequently, the extraction amount is reduced [59]. The effect of extraction duration on the total terpenoid yield was investigated. It was observed that the total terpenoid content decreased significantly when the extraction time exceeded 40 min. This phenomenon was attributed to the structural degradation of terpenoids during prolonged extraction, coupled with the increased dissolution of impurities that compromised the overall terpenoid yield. Additional experiments were conducted to evaluate the influence of temperature parameters on terpenoid extraction efficiency. The total terpenoids decreased as the temperature rose beyond 80 °C. This phenomenon may be credited with the deterioration of active compounds and a reduction in the extracted content, which occurs at elevated temperatures [54].

These compounds demonstrated stability in acids, bases, and organic solvents. They also exhibited effective separation and screening capabilities with minimal interference. Additionally, they showed strong adsorption capacity, easy regeneration potential, and a long service life. These properties provided unique advantages for the standardized isolation and commercial-scale production of natural products, making them widely applicable in natural product purification [60,61,62]. In addition, macroporous resin adhesion is a type of approach to purification using adsorbents that takes advantage of the differences between the adsorbent resin and the substance, and the nature of the functional clusters on the exterior of the adsorbent particles, such as polarity, acid–base, and hydrogen-bonding capacity, can impact the selectivity of adsorption and adsorption behavior [63]. As the fluid medium passed through the porous adsorbent bed, the reactive sites on solid-phase surfaces selectively interacted with fluid molecules [64]. The substance obtained from the preliminary extraction contained significant impurities (e.g., proteins and sugars), which affected the study of its physiological activity. Therefore, further separation and purification were required to remove these impurities and improve the purity of the target compound. In the current work, macroporous resin was employed for AATP purification. A screening of the available macroporous resins revealed that different resins exerted varying effects on the adsorption and desorption of total terpenoids. Based on the experimental findings, D101 packing was chosen for the purification of AATP. The optimal purification values were determined by dynamic sorption and desorption tests, i.e., a sample density of 0.8 mg/mL, a sample volume of 150 mL, and a sample flow speed of 1.0 mL/min. The elution solvent was 95% ethanol at a flow rate of 2.0 mL/min for a volume of 210 mL. The above conditions resulted in the improvement of the purity of AATP from 20.85% ± 0.94% before purification to 52.21 ± 0.75% after purification. Practical operation showed that this method is simple, low-cost, easy to promote, and is the best choice for the enrichment and purification of AATP.

It is now understood that atherosclerosis is an inflammatory condition with underlying mechanisms involving apoptosis, endothelial dysfunction, and oxidative stress [65]. It has been demonstrated that during the progression of atherosclerosis, LDL is reactive oxygen species-oxidized, leading to the production of oxidized LDL, which impairs endothelial cell function and activates the secretion of inflammatory cytokines by monocytes and macrophages to amplify inflammation [66]. Research had confirmed that natural terpenoids exhibited diverse pharmacological activities; therefore, it was speculated that AATP might possess anti-atherogenic properties. Experimental data demonstrated a significant inhibitory effect of AATP on atherosclerosis. Lipid metabolism disorders, as one of the key pathogenic factors in atherosclerosis, could be evaluated by measuring the serum levels of TC, TG, LDL-C, and HDL-C [67]. TC represented the total cholesterol content in blood lipoprotein particles and served as a risk predictor for atherosclerosis, while elevated TG levels were a contributing factor to atherosclerosis progression [68]. “Cholesterol hypothesis” was proposed, suggesting that lowering LDL cholesterol levels reduces the risk of atherosclerotic cardiovascular disease [69]. HDL-C can deliver cholesterol from plaque to the liver for catalysis, thus reversing cholesterol transit, reducing cholesterol deposition in the vessel wall, and playing an anti-atherosclerotic role [70]. The results suggested that the serum total cholesterol, total cholesterol, and LDL cholesterol levels were significantly reduced in AS rats after AATP intervention. This implies that AATP may play a therapeutic role in the treatment of atherosclerosis by regulating lipid metabolism disorders in AS rats. The development of lipid foam cells within atherosclerotic plaques is a pivotal process in the progression of lesions, including the deterioration of the tips of fibers, necrotic nuclei, and ultimately, plaque rupture. Foam cells are macrophages that overabsorb oxidized low-density lipoproteins. It is a distinct pathological cell type in atherosclerotic plaques [71]. It could be reasonably deduced that the inhibition of foam cell formation represented an efficacious therapeutic strategy at all stages of atherosclerosis, from early to advanced stages [72]. In this context, the authors investigated whether AATP inhibited lipid accumulation in macrophage-derived foam cells induced by ox-LDL. The results demonstrated that treatment with varying concentrations of AATP significantly reduced intracellular lipid droplet accumulation and suppressed macrophage foam cell formation.

In the treatment of atherosclerosis, a range of promising therapeutic strategies for inflammation modulation were proposed, including the inhibition of pro-inflammatory cytokines, the blockade of key inflammatory signaling pathways, and the promotion of inflammation resolution [73]. The microenvironment of atherosclerotic plaques is highly complex. Among the macrophage subtypes identified, there were anti-inflammatory macrophages (M2, M(Hb), Mhem, and Mox) and pro-inflammatory macrophages (M1 and M4). Various inflammatory responses and atherosclerotic plaque formation were triggered by an increase in pro-inflammatory macrophages and a decrease in anti-inflammatory macrophages, which accelerated the progression of cardiovascular diseases [74,75,76]. Numerous stimuli, such as lipopolysaccharides (LPSs) and interferon-γ (IFN-γ), activated M1 macrophages, leading to the production and release of pro-inflammatory cytokines (e.g., TNF-α, IL-1β, IL-6), as well as reactive oxygen species (ROS) and nitric oxide (NO) [77,78]. The alterations in these pro-inflammatory mediators were examined both in vivo and in vitro, and it was demonstrated that AATP significantly reduced the blood levels of TNF-α and IL-6 in AS rats and LPS-induced macrophages. The inhibitory effect of AATP on atherosclerosis was further confirmed by the combined analysis of biochemical indices and cellular experiments after modeling in rats. Additionally, AATP potently inhibited the production of NO and ROS in vitro. Furthermore, the effect of AATP on CD86 expression in LPS-induced macrophages was evaluated, and it was found that AATP significantly decreased CD86 expression, indicating its inhibition of LPS-induced polarization of RAW264.7 macrophages toward the M1 phenotype.

Since its initial proposal in 2007, network pharmacology has attracted significant interest in the fields of drug development and mechanism prediction. The rapid advancements in bioinformatics and integrative pharmacology facilitated cost-effective drug development through cyber pharmacology-based approaches. Molecular docking predicted ligand–receptor binding affinity and conformation [79,80,81]. Numerous studies have employed integrated strategies combining network pharmacology predictions with molecular docking. Based on network pharmacological analysis, the potential targets of AATP for atherosclerosis (AS) therapy include STAT3 and the JAK/STAT pathway, which are associated with inflammatory responses. Molecular docking demonstrated favorable interactions between JAK2/STAT3 pathway-associated proteins and the active ingredients of AATP. Therefore, it could be hypothesized that AATP exerts anti-inflammatory effects on atherosclerosis by inhibiting the JAK2/STAT3 signaling pathway. Recently, accumulating evidence has indicated that JAK2 phosphorylation is linked to cardiovascular conditions, including atherosclerosis, diabetes mellitus [82], myocardial fibrosis [83], and myocardial ischemia–reperfusion injury [84]. The regulation of JAK2 phosphorylation and its downstream pathways has the potential to ameliorate inflammation, making JAK2 a prospective therapeutic target for preventing and managing cardiovascular diseases (CVDs) [85]. The STAT3 dimer is rapidly translocated to the nucleus, ultimately regulating the expression of downstream genes at the transcriptional level [86]. The JAK2/STAT3 signaling pathway is a classical inflammatory signaling pathway that regulates macrophage polarity toward the M1 phenotype and modulates the secretion of inflammatory factors (e.g., TNF-α), which exacerbates the course of atherosclerosis [87,88]. Several studies have demonstrated that phytochemicals and natural plants exert a protective effect against cardiovascular diseases and regulate JAK2/STAT3 signaling [89,90,91]. Evidence that AATP inhibits LPS-triggered JAK2-STAT3 phosphorylation in macrophages suggests that its anti-atherosclerotic effects may involve the modulation of this proinflammatory signaling axis. Clinical drug interventions primarily focus on reversing coronary atherosclerotic plaques, with lipid-lowering drugs, especially statins and PCSK9 inhibitors, remaining central to treatment because they not only lower cholesterol but also have anti-inflammatory and plaque-stabilizing properties [92]. Compared to single compounds, natural extracts contain multiple active ingredients, and the synergistic effects of each component through “multi-target-multi-pathway” enhance the therapeutic effect. After long-term verification through folk medicine, the toxicity and side effects are generally lower than those of synthetic drugs. The cost-effectiveness ratio is high. The extraction and purification process is relatively simpler than chemical synthesis (especially crude extract preparations).

This study conducted a detailed investigation into the extraction and purification process of AATP. Subsequent research can focus on developing more terpenoid active components in AATP, with an emphasis on their specific active effects. AATP exerts its anti-atherosclerotic effects by inhibiting lipid accumulation and inflammatory responses in RAW264.7 foam cells. Further research can be conducted to identify additional targets of AATP in atherosclerosis and to explore its anti-atherosclerotic mechanisms in greater depth, thereby providing a promising natural drug for the treatment of atherosclerosis.

## 5. Conclusions

In conclusion, we determined the preferred extraction process for AATP, purified the extracted AATP using macroporous resin, and investigated the optimal conditions for purification. The anti-atherosclerotic effects of AATP were investigated in vitro and in vivo by identifying the targets of AATP in atherosclerosis through network pharmacology and molecular docking. AATP exhibited potent antioxidant and anti-inflammatory activities, which were related to its inhibition of the expression of JAK2/STAT3 pathway-related proteins. AATP attenuated the aortic and hepatic lesions in rats with AS, thereby exerting an anti-atherosclerotic effect. The results of this study provide an experimental basis for the further exploration of more targets of AATP against atherosclerosis and a deeper investigation into its anti-atherosclerotic mechanism, and the results also suggest a promising natural drug for atherosclerosis.

## Figures and Tables

**Table 1:** Factors and levels in response surface design.

Levels	Material–Liquid Ratio (A) (g/mL)	Extraction Temperature (B) (°C)	Extraction Time (C) (min)
−1	20	70	30
0	25	80	40
1	30	90	50

**Table 2:** Analytical factors and levels for BBD.

Test Number	Material–Liquid Ratio (A)	Extraction Temperature (B)	Extraction Time (C)	Total Terpenoid Content (mg/g)
1	30	80	30	13.63
2	25	80	40	18.39
3	25	70	30	14.26
4	25	80	40	18.26
5	25	90	30	14.53
6	30	80	50	15.11
7	25	90	50	14.77
8	20	80	50	13.05
9	30	70	40	13.61
10	30	90	40	14.58
11	20	80	30	14.02
12	20	90	40	14.17
13	25	80	40	17.61
14	25	80	40	18.26
15	25	70	50	14.28
16	25	80	40	18.13
17	20	70	40	13.77

**Table 3:** ANOVA results of the response model.

Source	Sum of Squares	Df	Mean Square	F Value	*p*-Value	Significance	95%Confidence Interval	
							**Lower Bound**	**Upper Bound**
Model	54.95	9	6.11	56.28	<0.0001 **	significant		
A	0.46	1	0.46	4.25	0.0782		−0.0353	0.5153
B	0.56	1	0.56	5.23	0.0561		−0.0091	0.5416
C	0.074	1	0.074	0.68	0.4357		−0.1791	0.3716
AB	0.081	1	0.0812	0.74	0.4155		−0.2469	0.5319
AC	1.50	1	1.50	13.83	0.0075 **		0.2231	1.00
BC	0.012	1	0.012	0.11	0.7482		−0.3344	0.4444
A2	20.84	1	20.84	192.08	<0.0001 **		−2.60	1.84
B2	12.41	1	12.41	114.44	<0.0001 **		−2.01	−1.34
C2	13.06	1	13.60	125.35	<0.0001 **		−2.18	−1.42
Lack of Fit	0.3064	3	0.10	0.90	0.5144	not significant		
Pure Error	0.452	4	0.01132					
Cor Total	55.71	16						
R2	0.9864							
R2adj	0.9688							

**Table 4:** Comparison of the static adsorption and desorption properties of different macroporous resins.

Resin Type	HPD600	D101	AB-8	NKA-9	DM130	X-5
Polarity category	Polarity	Non-polar	Weak polarity	Polarity	Weak polarity	Non-polar
Specific surface area/(m^2^/g)	550	500–550	480–520	250–290	500–550	500–600
Average aperture/nm	10~12	9~11	13~14	10~12	9~10	29~30
Moisture content%	73.5	67.50	71.64	72.50	74.20	71.00
Adsorption capacity mg/g	5.72 ± 0.15	7.25 ± 0.22	7.04 ± 0.23	6.55 ± 0.14	6.29 ± 0.20	7.09 ± 0.14
Desorbent capacity mg/g	1.71 ± 0.20	2.58 ± 0.095	2.18 ± 0.010	1.75 ± 0.044	2.02 ± 0.080	2.27 ± 0.012

## Data Availability

The original contributions presented in the study are included in the article, further inquiries can be directed to the corresponding authors.

## Abbreviations

The following abbreviations are used in this manuscript:

AATP	Total terpenoids of *Arlemisia absinthtum* L.;
AS	Atherosclerosis;
CVD	Cardiovascular disease;
RSM	Response surface methodology;
HFD	High-fat diet;
OX-LDL	Oxidized low-density lipoprotein;
LPS	Lipopolysaccharide;
BBD	Box–Behnken design;
ROS	Reactive oxygen species;
TC	Total cholesterol;
TG	Triglyceride;
TNF	Tumor necrosis factor;
IL	Interleukin;
JAK	Janus Kinase;
STAT	Signal transducer and activator of transcription;
LDL-C	Low-density-lipoprotein cholesterol;
HDL-C	High-density-lipoprotein cholesterol;
